# Space-Confined Effect One-Pot Synthesis of γ-AlO(OH)/MgAl-LDH Heterostructures with Excellent Adsorption Performance

**DOI:** 10.1186/s11671-019-3112-x

**Published:** 2019-08-16

**Authors:** Guoyuan Zheng, Caihong Wu, Jilin Wang, Shuyi Mo, Zhengguang Zou, Bing Zhou, Fei Long

**Affiliations:** 1 Key Laboratory of New Processing Technology for Nonferrous Metals and Materials of Ministry of Education, Guilin University of Technology, Guilin, 541004 China; 2 Guangxi Key Laboratory of Optical and Electronic Materials and Devices, Guilin University of Technology, Guilin, 541004 China; 3 Collaborative Innovation Center for Exploration of Hidden Nonferrous Metal Deposits and Development of New Materials in Guangxi, Guilin University of Technology, Guilin, 541004 China

**Keywords:** γ-AlO(OH)/MgAl-LDH, Space-confined, Langmuir model, Potential adsorbent

## Abstract

Herein, γ-AlO(OH) as an inorganic was successfully inserted into MgAl-LDH layer by a one-pot synthesis, the composite as an adsorbent for removing methyl orange (MO) from wastewater. The structure and adsorption performance of γ-AlO(OH)/MgAl-LDH were characterized. The research shows that the expansion (003) plane and the hydroxyl active site of γ-AlO(OH)/MgAl-LDH can promote adsorption capacity and adsorption kinetics, respectively. Therefore, γ-AlO(OH)/MgAl-LDH exhibits a super adsorption performance, which completely adsorbs MO at the concentration of 1000 mg g^−1^. In addition, the maximum adsorption capacity of MO was 4681.40 mg g^−1^ according to the Langmuir model. These results indicate that γ-AlO(OH)/MgAl-LDH is a potential adsorbent for the removal of organic dyes in water.

## Introduction

Organic dyes are widely used in many products such as textiles, leather, paint, and rubber [1–3]. These dyes are easily discharged into water [4], causing serious environmental problems such as harming aquatic organisms, consuming dissolved oxygen, and staining water [3, 5]. In addition, most organic dyes are highly polar, nonvolatile, and difficult to biodegrade. It is observed that dye wastewater is severely detrimental to human health. Therefore, the treatment of dye wastewater is an urgent task. At present, most of the treatments for dye wastewater use physical adsorption, photocatalysis, biological and chemical oxidation, flocculation, and membrane separation [4, 6]. Among them, the physical adsorption method has a special position in the field of wastewater treatment due to its ability to selectively enrich certain compounds. In addition, the adsorption method has the characteristics of a good adsorption effect, simple operation, and wide application range and has been widely used in the field of dye wastewater treatment [7, 8].

Layered double hydroxides (LDHs), a common anionic clay, consist of brucite-like layers [9]. Its general formula can be expressed as [*M*^2+^_1 − *x*_*M*^3+^_*x*_(OH)_2_][(*A*^*n*−^)_*x*/*n*_]·yH_2_O, where *M*^2+^, *M*^3+^, and *A*^*n*−^ represent the bivalent cations, trivalent cations, and *n*-valent anions, respectively [10]. LDH has excellent adsorption properties for dyes due to its high anion exchange capacity and large surface area. For example, Lafi et al. prepared MgAl-LDH by a coprecipitation method; the adsorption capacity of the adsorbent on Congo red reached 111.111 mg g^−1^ [11]. Zheng et al. prepared Zn-Mg-Al LDH also via the coprecipitation method, which have an excellent adsorption capacity as high as 883.24 mg g^−1^ for methyl orange at the condition of pH = 3 [12]. Clearly, for LDH adsorbents, most researchers focus on the pursuit of high ion exchange capacity and large specific surface area. Unfortunately, the nanocrystallization of LDHs is not without limits. In recent years, researchers have found that organic or inorganic insert to LDH layers can increase the adsorption capacity of LDH. For example, Mandal et al. inserted sodium alginate between the LDH layers to form a composite adsorbent. The sodium alginate helps in widening the interlayer space of the LDH and increases the adsorption capacity of the adsorbent for the orange II dye [13]. Bruna et al. synthesized organic/LDH (organic anion dodecylsulfate (DDS) insert to MgAl-LDH) as an adsorbent of polycyclic aromatic hydrocarbons in water and soil-water systems [14]. Therefore, it is a good idea to design the adsorbent by inserting a compound between the LDH layers. Aluminum oxide hydroxide (γ-AlO(OH)) is a good adsorbent for wastewater treatment, due to high specific surface area and large amount of hydroxyl groups on the surface [15, 16]. Hence, γ-AlO(OH) is a potential inorganic intercalation material for MgAl-LDH.

In this paper, γ-AlO(OH) was successfully inserted to MgAl-LDH by a hydrothermal method. This composite exhibits excellent adsorption properties for methyl orange (MO). The structural characteristics of γ-AlO(OH)/MgAl-LDH composites were evaluated using X-ray powder diffraction (XRD), Fourier-transform infrared spectroscopy (FTIR), field-emission scanning electron microscopy (FESEM), transmission electron microscopy (TEM), and high-resolution transmission electron microscopy (HRTEM). The adsorption properties of the composites were evaluated via the adsorption of MO, and in-depth research on the synergistic mechanism of γ-AlO(OH) and MgAl-LDH was conducted.

## Methods

### Preparation of γ-AlO(OH)/MgAl-LDH

All chemical reagents were of analytical grade and used without further purification. The γ-AlO(OH)/MgAl-LDH composite was prepared using the hydrothermal method. In a typical synthesis, Mg(NO_3_)_2_·6H_2_O (4.615 g) and Al(NO_3_)_3_·9H_2_O (3.376 g) were dissolved in 50 mL of deionized (DI) water (Mili-Q, 18.2 MΩ) to form solution 1. NaOH (2.516 g) was dissolved in 25 mL of degassed deionized water, such that solution 2 was produced. Solutions 1 and 2 were added dropwise to a reaction vessel containing 25 mL of deionized water, and stirring was performed vigorously at a constant pH value of 10 and a temperature of 60 °C. Then, the resultant slurry was further treated under a hydrothermal condition at 140 °C for 10 h and cooled to room temperature. γ-AlO(OH)/MgAl-LDH was washed several times with deionized water and lyophilized in a vacuum freeze dryer. For comparison, pure MgAl-LDH and γ-AlO(OH) were prepared by the same hydrothermal treatment (140 °C, 10 h).

### Characterization

The phase structure was characterized by powder X-ray diffraction (XRD; X’Pert PRO PANalytical) in the 2*θ* range of 5–80° with Cu Kα radiation at a wavelength of 0.15406 nm. The surface morphology of the sample was imaged by FESEM (S4800) at 5 kV. The microstructure of the samples was analyzed by HRTEM (JEM-2100F) at 200 kV. IR spectra were recorded in the range of 4000–400 cm^−1^ using a FTIR spectrometer (NEXUS 470, Nicolet instruments) with an optical resolution of 4 cm^−1^ and an aperture size of 100 im. Nitrogen adsorption-desorption experiments for surface and porosity quantification were performed at − 196 °C with a NOVA-1200e instrument. Prior to analysis, samples were pretreated at 80 °C for 12 h under vacuum. X-ray photoelectron spectroscopy (XPS; ESCALAB 250Xi) measurements were performed using Al Kα radiation. The energy of survey spectrum scanning was 100 eV by the step size of 1 eV. The energy of high-resolution scanning was 20 eV by the step size of 0.1 eV. The vacuum of the test is 10^−10^ mbar. The UV–Vis absorption spectra of the different samples were obtained using a UV-3600 spectrophotometer equipped with an integrating sphere. The photoluminescence spectra of the materials were obtained by a fluorescence spectrophotometer (VARIAN).

### Adsorption Experiments

The adsorption performance of the samples was tested for the adsorption of methyl orange (MO) in aqueous solution. A 50-mg sample was placed in 50 mL of 1000 mg L^−1^ MO solution under magnetic stirring. The pH value of the solution was adjusted using 0.1 M HNO_3_ acid or 1 M NaOH solution. After an appropriate time, the water sample (3 mL) was taken from the suspension. The supernatant was obtained by centrifugation, and the concentration of the solution was measured using a UV–Vis spectrophotometer (UV-3600). The equilibrium amount of adsorption (*q*_*e*_ (mg g^−1^)) and the instantaneous amount of adsorption (*q*_*t*_ (mg g^−1^)) were calculated by the following equations:

$$ {q}_t=\frac{\left({C}_0-{C}_t\right)V}{m}\kern35em (1) $$

$$ {q}_e=\frac{\left({C}_0-{C}_e\right)V}{m}\kern35.25em (2) $$

where *C*_0_ (mg L^−1^) is the initial MO concentration; *C*_*e*_ (mg L^−1^) and *C*_*t*_(mg L^−1^) are the MO concentration at equilibrium and at time *t* (min), respectively; *V* (L) is the volume of the solution; and *m* (g) is the mass of the adsorbent.

### Desorption Experiments

The desorption experiment of MO was performed using DI water as a disturbing agent. A 50-mg portion of the used samples was washed gently with water to remove any undisturbed MO. In addition, loaded MO samples were stirred vigorously with ethanol solution and centrifuged. After centrifugation, the obtained samples were lyophilized. Then, the resulting powder samples were subjected to successive adsorption-desorption cycles.

## Results and Discussion

### Characterization of As-Synthesized Samples

**Fig. 1:**
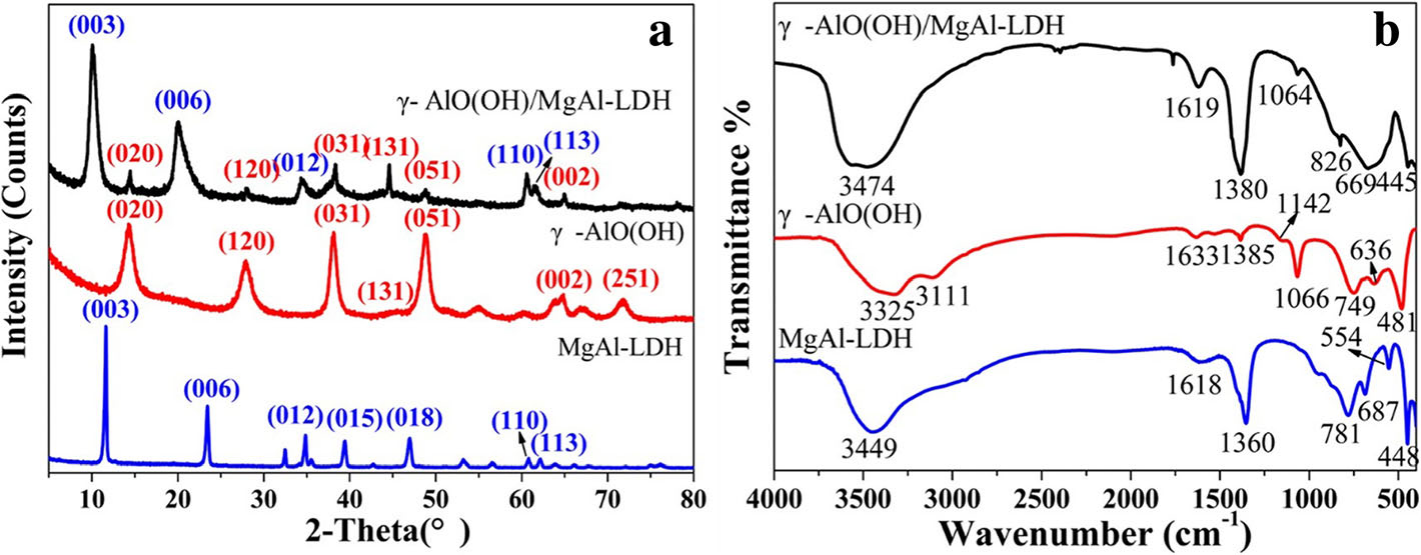
XRD patterns of as-synthesized samples (**a**). The FTIR spectra of as-synthesized samples at 400–4000 cm^−1^ (**b**)

The XRD patterns of the as-synthesized samples are shown in Fig. 1a. For γ-AlO(OH)/MgAl-LDH, it is observed that the major diffraction peaks are at 10.09°, 19.95°, 34.40°, 60.56°, and 61.48°, which corresponded to the (003), (006), (012), (110), and (113) planes of MgAl-LDH (JPCDS No. 89-0460), respectively. In addition, the peaks at 14.1°, 27.9°, 38.1°, and 48.9° can be attributed to the (020), (120), (031), and (051) diffraction planes of γ-AlO(OH) (JPCDS No. 21-1307), respectively. This result indicates that the γ-AlO(OH)/MgAl-LDH composite has MgAl-LDH and γ-AlO(OH) phases. Moreover, for comparison, the (003) plane of MgAl-LDH is located at 2*θ* = 11.63°, indicating that when γ-AlO(OH) was inserted into MgAl-LDH, the spacing of the (003) plane increased from 7.6 Å (2*θ* = 11.63°) to 8.77 Å (2*θ* = 10.09°). The unit cell parameters are shown in Table 1. It is observed that the “a” axes of MgAl-LDH and γ-AlO(OH)/MgAl-LDH have not changed.

**Table 1:** Unit cell parameters, crystallite size, surface area, pore volume, and average pore diameter of the samples

Sample	Unit cell parameters (Å)		Crystallite size^a^ (nm)		Surface area^b^ (m^2^ g^−1^)	Pore volume^b^ (cm^3^ g^−1^)	Average pore size^b^ (nm)
	a	c	D_003_	D_110_			
MgAl-LDH	3.05	22.8	31.0	26.6	14.1	0.017	5.2
γ-AlO(OH)	n.a.	n.a.	n.a.	n.a.	95.9	0.122	4.9
γ-AlO(OH)/MgAl-LDH	3.05	26.3	10.3	26.6	34.1	0.052	6.2

a = 2d_110_, c = 3d_003_

^a^Obtained from the Debye-Scherrer formula

^b^Calculated from the BET analysis

The FTIR spectra of the as-synthesized samples are shown in Fig. 1b. In the FTIR spectrum of MgAl-LDH, the band at 3449 cm^−1^ can be attributed to the O–H stretching vibration [17]. The bands from 400 to 900 cm^−1^ are due to M–O, O–M–O, and M–O–M (M = Mg^2+^ and Al^3+^) stretching vibrations [18], and the band at 781 cm^−1^ is due to an Al–OH vibration [19]. For γ-AlO(OH), the bands at 3111 and 3325 cm^−1^ belong to the υas(Al)O–H and υs(Al)O–H stretching vibrations, respectively [20]. The vibrations of hydrogen bonds were observed at 1142 and 1066 cm^−1^ [21]. In addition, the peaks at 481, 636, and 749 cm^−1^ can be assigned to Al–O bonds [22]. For γ-AlO(OH)/MgAl-LDH, most of the bands can be easily assigned based on the comparison with γ-AlO(OH) and MgAl-LDH. The strong band at 3474 cm^−1^ can be attributed to the stretching vibration of the –OH groups in γ-AlO(OH) and MgAl-LDH. The bands at 826, 669, and 445 cm^−1^ correspond to metal-oxygen, metal-oxygen-metal, and oxygen-metal-oxygen stretching vibrations in MgAl-LDH, respectively [18]. Compared with MgAl-LDH, the band of Al–OH is shifted from 781 to 826 cm^−1^. In addition, the band at 1064 cm^−1^ can be assigned to the vibration of the hydrogen bond in γ-AlO(OH). The bands at 1618, 1633, and 1619 cm^−1^ in MgAl-LDH, γ-AlO(OH), and γ-AlO(OH)/MgAl-LDH, respectively, can be assigned to the bending vibration of the water molecules. In addition, the bands at 1360, 1385, and 1380 cm^−1^ in MgAl-LDH, γ-AlO(OH), and γ-AlO(OH)/MgAl-LDH, respectively, are related to CO_3_^2−^ [23]. The band of CO_3_^2−^ in γ-AlO(OH) indicates that some carbonate-based residuals remain trapped inside the highly porous cellular monolith even after repeated washing [24].

**Fig. 2:**
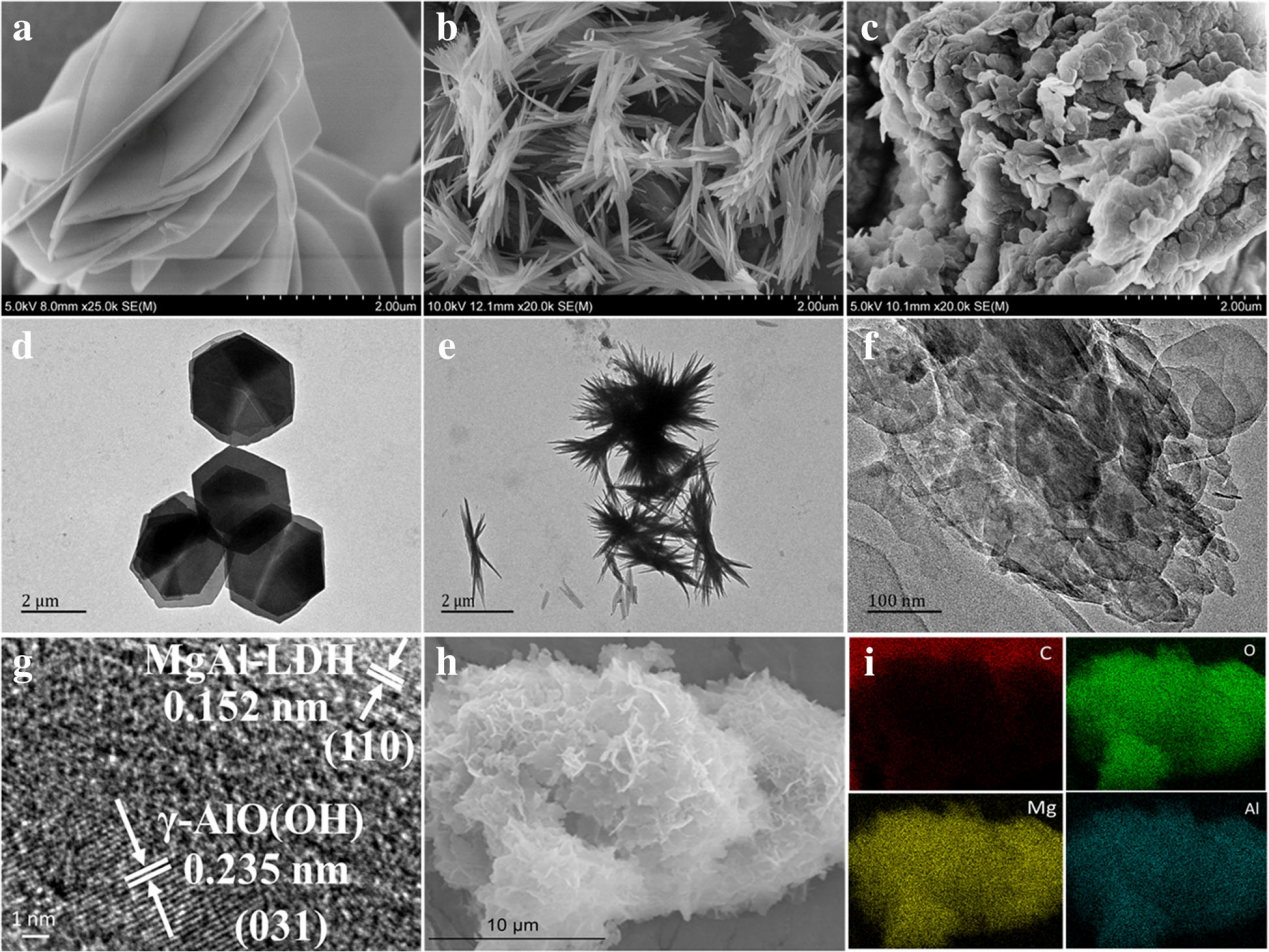
FESEM images of MgAl-LDH (**a**), γ-AlO(OH) (**b**), and γ-AlO(OH)/MgAl-LDH (**c**). TEM images of MgAl-LDH (**d**), γ-AlO(OH) (**e**), and γ-AlO(OH)/MgAl-LDH (**f**). HRTEM image (**g**) and EDX mapping (**h**, **i**) of γ-AlO(OH)/MgAl-LDH

The morphology and microstructure of the samples were investigated by field-emission scanning electron microscopy (FESEM) and transmission electron microscopy (TEM). As shown in Fig. 2a, it is observed that the MgAl-LDH sample consists of nanosheets. The average thickness of the nanosheets is estimated to be between 140 and 150 nm. The FESEM image in Fig. 2b shows that γ-AlO(OH) consists of nanoneedles. The γ-AlO(OH)/MgAl-LDH sample shown in Fig. 2c has a morphology consisting of flaky agglomerates rather than the morphology of γ-AlO(OH). In the case of Fig. 2d, e, the TEM images of MgAl-LDH and γ-AlO(OH), respectively, also show strong evidence of the nanosheet-like morphology of MgAl-LDH and the nanoneedle-like morphology of γ-AlO(OH). Interestingly, it is clearly observed that the γ-AlO(OH)/MgAl-LDH sample consists of nanosheets and nanoneedles (Fig. 2f). In addition, in the HRTEM image of γ-AlO(OH)/MgAl-LDH (Fig. 2g), the lattice spacing 0.235 nm and 0.152 nm corresponded to the (031) plane of γ-AlO(OH) and (110) plane of MgAl-LDH. In addition, EDX mapping of Fig. 2h, i demonstrated the uniform distribution of C, O, Mg, and Al elements in composites, indicating that γ-AlO(OH)/MgAl-LDH composite was mixed homogeneously.

The XRD, FTIR, FESEM, and HRTEM analyses show that the nanoneedle γ-AlO(OH) was successfully prepared in the MgAl-LDH layers by the hydrothermal method, which takes advantage of the “space-confined” effect of MgAl-LDH.

### Effect of the Initial Solution pH

**Fig. 3:**
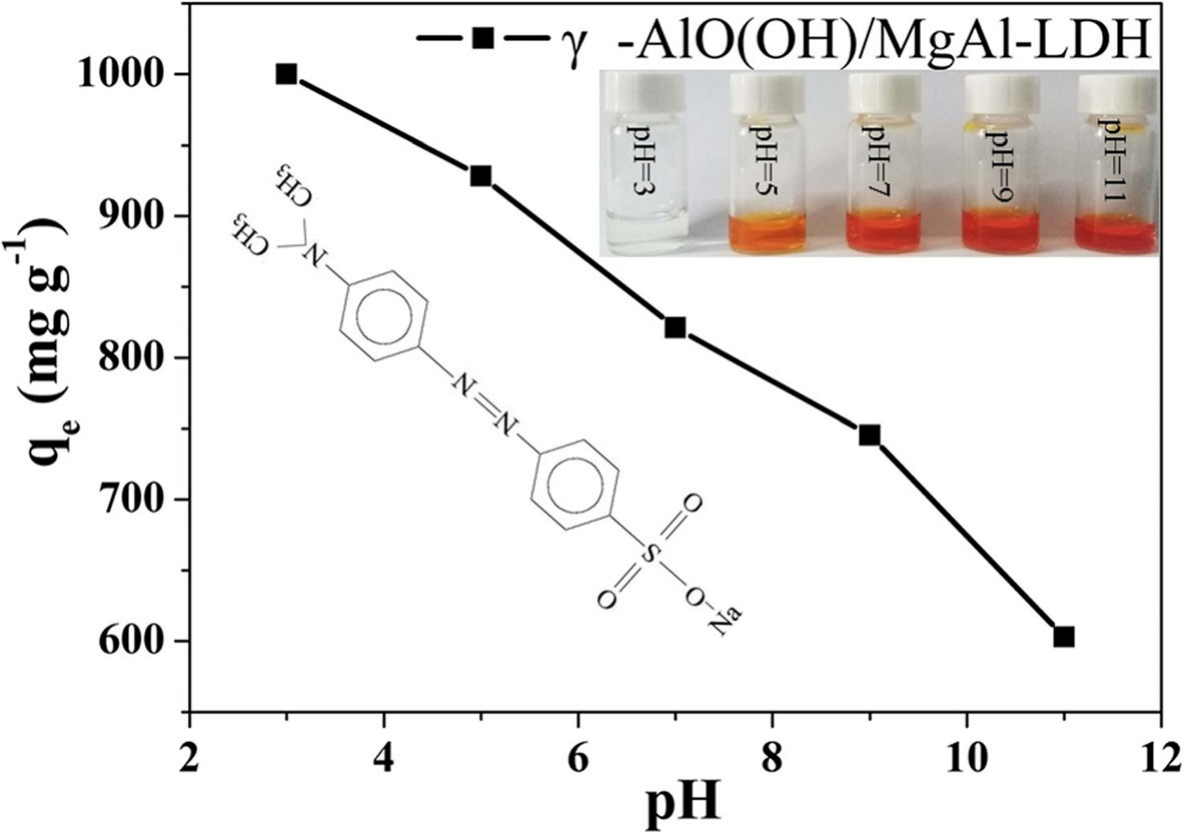
Effect of initial solution pH values on the adsorption of MO of γ-AlO(OH)/MgAl-LDH (initial MO concentration: 1000 mg L^−1^, adsorbent dosage: 1 g L^−1^, contact time: 210 min), inset: the optical photographs of MO solution after adsorbed at different pH

The pH of the solution plays an important role in the adsorption process due to the surface charge of the adsorbent [25]. Figure 3 shows the adsorption performance of the γ-AlO(OH)/MgAl-LDH sample on MO under different pH values, with the initial MO concentration at 1000 mg L^−1^. It is observed that the highest adsorption occurs when the initial pH = 3, and the adsorption capacity decreases with increasing pH, indicating that the as-synthesized sample is more effective at adsorbing MO in acidic solution. Furthermore, the structures of the layered materials with hydroxide sheets vanish when the pH is below 3 [26]. Therefore, the initial solution pH used in this study is suggested to be 3. Photographs of the γ-AlO(OH)/MgAl-LDH sample adsorbing MO at different pH values are also shown in Fig. 3 (inset). It is observed that at pH = 3, after MO was adsorbed for 210 min, the color of the solution was clear, indicating that MO was completely adsorbed. As the pH increased, the color of the solution became darker.

### Effect of the Contact Time and the Kinetics of Adsorption

**Fig. 4:**
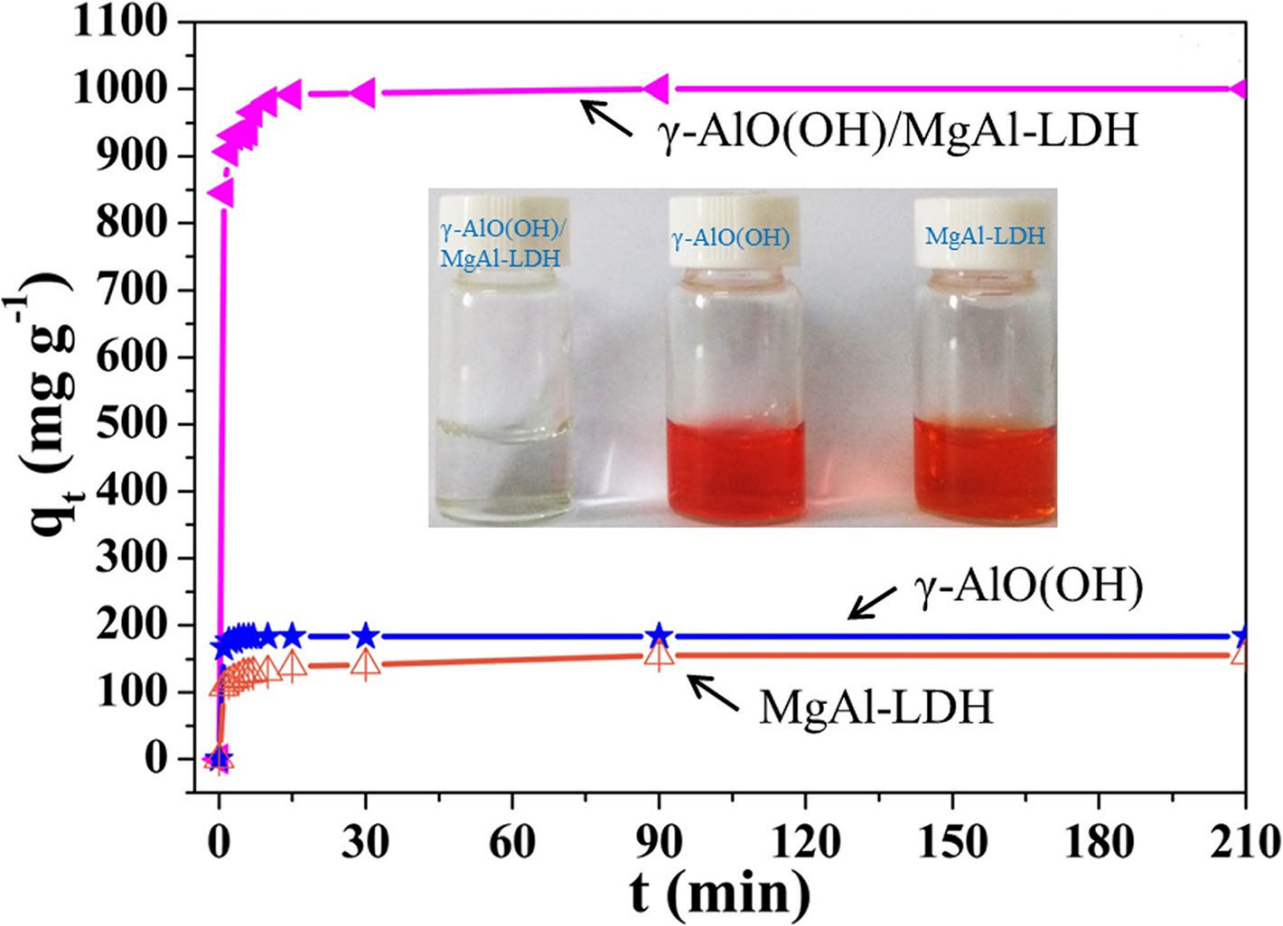
Effect of contact time on MO adsorption, inset: the equilibrium optical photographs of MO solution after adsorbed

The effect of the contact time on the MO adsorption by the samples is shown in Fig. 4. For all adsorbents, the initial rates of adsorption are very fast. Compared with pure γ-AlO(OH) and MgAl-LDH, γ-AlO(OH)/MgAl-LDH shows an enhanced adsorption performance in terms of the adsorption speed and capacity. When the initial concentration of MO was 1000 mg L^−1^ for γ-AlO(OH)/MgAl-LDH and 200 mg L^−1^ for γ-AlO(OH) and MgAl-LDH, the maximum experimental equilibrium adsorption capacity of 1000 mg g^−1^ was obtained using γ-AlO(OH)/MgAl-LDH, which was higher than that of γ-AlO(OH) (183.3 mg g^−1^) and MgAl-LDH (155.5 mg g^−1^). As shown in the Fig. 4 (inset), it is observed that the γ-AlO(OH)/MgAl-LDH solution is completely colorless after equilibration. However, the colors of the pure γ-AlO(OH) and MgAl-LDH solutions remain very deep.

To understand the adsorption mechanisms of the samples, kinetic models of pseudo-first-order and pseudo-second-order were used to fit the experimental data. The pseudo-first-order and pseudo-second-order rate laws are calculated from Eqs. (3) and (4) [27], respectively:

$$ \ln \left({q}_e-{q}_t\right)=\ln {q}_e-{k}_1t\kern30.25em (3) $$

$$ \frac{t}{q_t}=\frac{1}{k_2{q}_e^2}+\frac{t}{q_e}\kern37.25em (4) $$

where *q*_*t*_ (mg g^−1^) and *q*_*e*_ (mg g^−1^) are the amount of adsorbed MO at time *t* (min) and at equilibrium, respectively, and *k*_1_ (min^−1^) and *k*_2_ (g mg^−1^ min^−1^) are the adsorption rate constants of the pseudo-first-order and pseudo-second-order kinetic models [28], respectively.

Table 2 shows the two adsorption kinetic models and correlation coefficients. From the correlation coefficient *R*^2^ in Table 2, it is observed that the adsorption of samples was fitted better by the pseudo-second-order model than by the pseudo-first-order model. In addition, the theoretical calculated values (*q*_e,cal_) from the pseudo-second-order model are closer to the experimental values (*q*_e,exp_) than those of the pseudo-first-order model. Therefore, based on the assumption of pseudo-second-order kinetics, the adsorption rates of MgAl-LDH, γ-AlO(OH), and γ-AlO(OH)/MgAl-LDH are controlled by chemical interactions [29].

**Table 2:** Kinetic parameters for the adsorption of MO onto MgAl-LDH, γ-AlO(OH), and γ-AlO(OH)/MgAl-LDH

Kinetic models	Parameters	MgAl-LDH	γ-AlO(OH)	γ-AlO(OH)/MgAl-LDH
Pseudo-first-order	*q*_e_ (mg g^−1^) experiment	155.5	183.3	1000
	*q*_e_ (mg g^−1^) model	48.6	40.9	146.7
	*K*_1_ (min^−1^)	0.05537	0.64079	0.13575
	*R* ^2^	0.50311	0.86471	0.65129
Pseudo-second-order	*q*_e_ (mg g^−1^) model	156.5	183.5	1001.3
	*K*_2_ (g mg^−1^ min^−1^)	0.00523	0.11053	0.00450
	*R* ^2^	0.9998	1	1

### Adsorption Isotherms

**Fig. 5:**
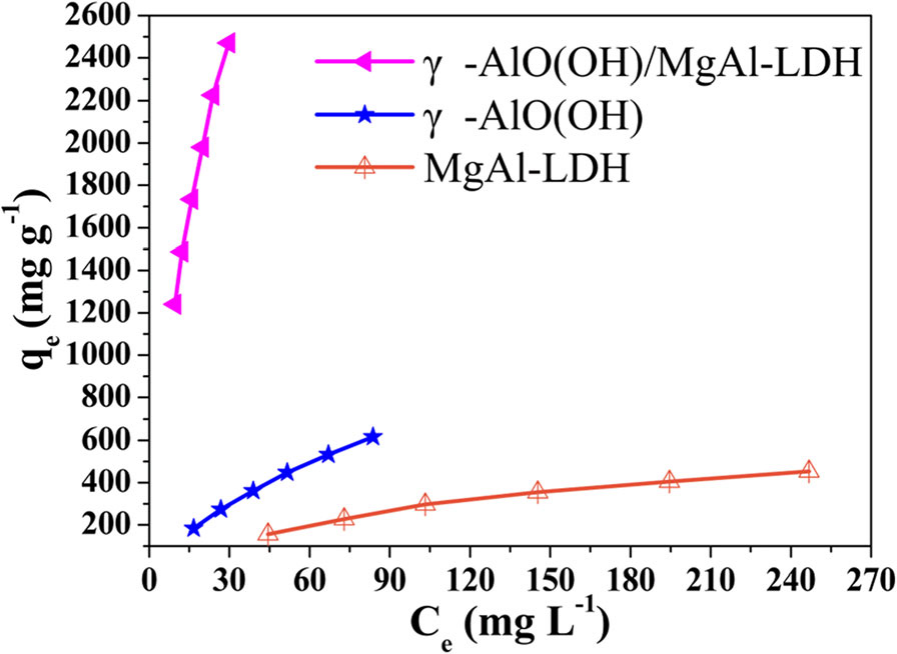
The equilibrium adsorption isotherms of the samples

Figure 5 shows the adsorption isotherms of the as-synthesized samples. Among the three samples, the *q*_*e*_ value of γ-AlO(OH)/MgAl-LDH exhibited the fastest increase with *C*_*e*_. In addition, the adsorption experiment data were analyzed via the Langmuir and Freundlich models to evaluate the relationship between MO and adsorbents at equilibrium [30]. The equations are as follows:

$$ \mathrm{Langmuir}:\frac{C_e}{q_e}=\frac{1}{q_m{K}_L}+\frac{C_e}{q_m}\kern29em (5) $$

$$ \mathrm{Freundlish}:\ln {q}_e=\ln {K}_F+\frac{1}{n}\ln {C}_e\kern27.25em (6) $$

where *C*_*e*_ (mg L^−1^) is the equilibrium concentration of the MO, *q*_*e*_ (mg g^−1^) is the amount of MO adsorbed per unit mass of the adsorbents, and *q*_*m*_ (mg g^−1^) is the monolayer adsorption capacity. *K*_*L*_ (L mg^−1^) is the Langmuir constant related to the rate of adsorption. *K*_*F*_ [(mg g^−1^) (L mg^−1^)^1/*n*^] and *n*^−1^ (dimensionless) are Freundlich constants related to the adsorption capacity and adsorption strength, respectively.

The corresponding parameters of the simulated adsorption isotherms of the samples are listed in Table 3. It is observed that the maximum adsorption capacity of MO on γ-AlO(OH)/MgAl-LDH was significantly higher than that on γ-AlO(OH) and MgAl-LDH. Moreover, compared to the Freundlich model, the Langmuir model fits better with the experimental data and has a higher *R*^2^. The *q*_m_ of γ-AlO(OH)/MgAl-LDH (4681.40 mg g^−1^) from the Langmuir equation is much higher than those of γ-AlO(OH) (1492.5 mg g^−1^) and MgAl-LDH (769.2 mg g^−1^). In addition, the larger *K*_F_ from the Freundlich equation also indicates that γ-AlO(OH)/MgAl-LDH has an enhanced affinity for MO. Surprisingly, the adsorption capacity of γ-AlO(OH)/MgAl-LDH was much higher than most of those reported (as shown in Table 4).

**Table 3:** Parameters of two types of isothermal models of MO adsorbed on MgAl-LDH, γ-AlO(OH), and γ-AlO(OH)/MgAl-LDH

Isothermal models	Parameters	MgAl-LDH	γ-AlO(OH)	γ-AlO(OH)/MgAl-LDH
Langmuir	*K*_L_ (L mg^−1^)	0.00582	0.00830	0.03741
	*q*_m_ (mg g^−1^)	769.2	1492.5	4681.4
	*R* ^2^	0.99648	0.9977	0.99639
Freundlich	*K*_F_ [(mg g^−1^)(L mg^−1^)^1/n^]	15.6	22.6	319.0
	*n* ^−1^	0.62077	0.75255	0.60843
	*R* ^2^	0.98187	0.99748	0.99563

**Table 4:** Comparison of the maximum adsorption capacity for MO with other adsorbents

Adsorbents	pH	*q*_m_ (mg g^−1^)	References
ZnAl-LDO	6.0	181.90	[31]
CoFe_2_O_4_/MgAl-LDO	6.0	1219.51	[2]
Graphene oxide	3.0	16.83	[32]
Chitosan/Al_2_O_3_/magnetite composite	6.0	416.00	[33]
rGO/Ni/MMO	n.a.	210.80	[7]
Ni-Cr LDH	n.a.	312.50	[34]
Mg_2_/Fe-CLDH	n.a.	194.90	[35]
G-LDH	4.5	443.50	[8]
G-LDO	4.5	1062.30	[8]
γ-AlO(OH)/MgAl-LDH	3.0	4681.40	This work

### The Adsorption Mechanism of γ-AlO(OH)/MgAl-LDH for MO

**Fig. 6:**
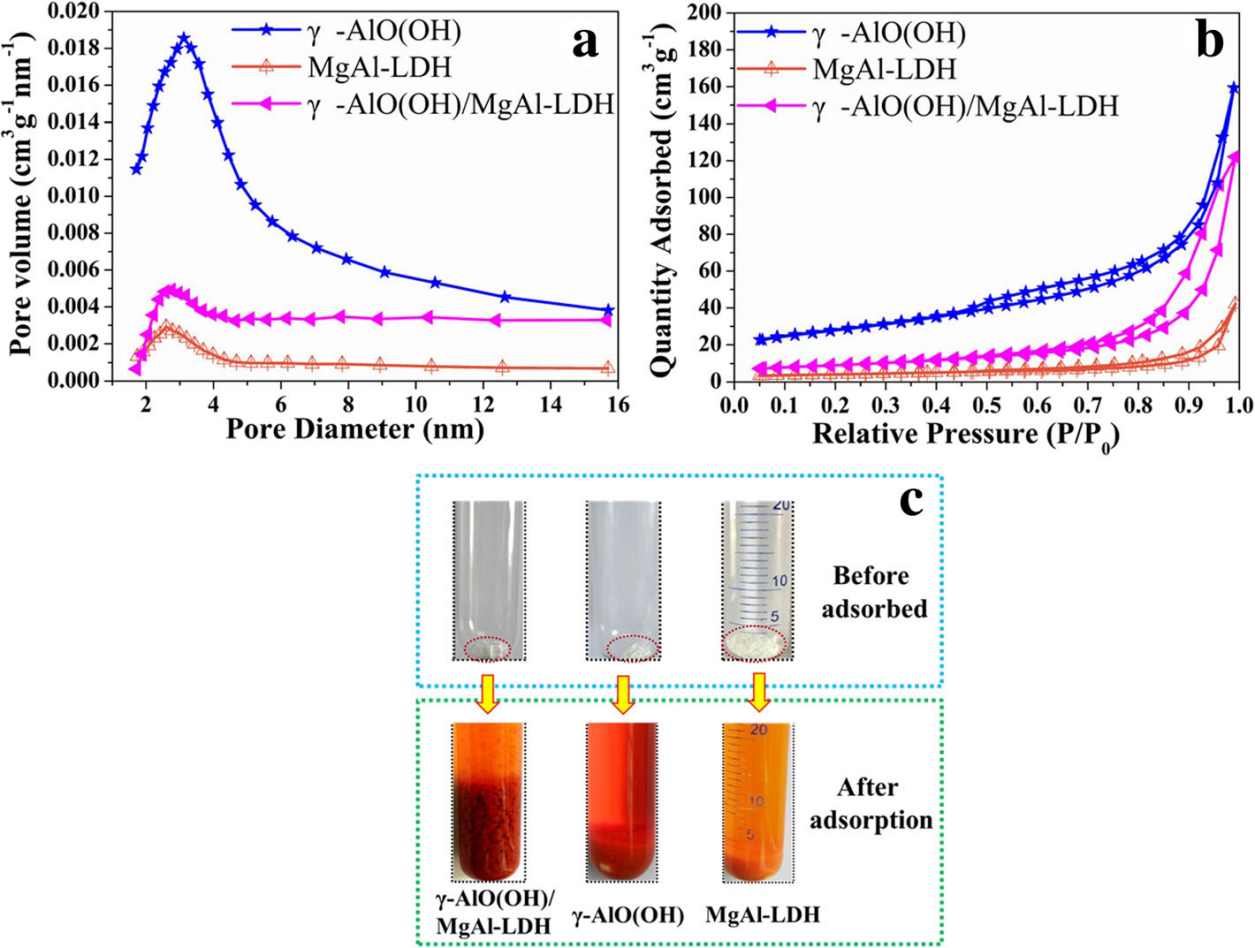
N_2_ adsorption-desorption isotherm curves (**a**) and pore size distribution curves (**b**) of samples. The optical photographs of samples before and after adsorbed (**c**)

Figure 6a, b shows the N_2_ adsorption-desorption isotherms and BJH pore size distribution of samples. According to the IUPAC classification, the isotherms can be categorized as IV curves with H3 hysteresis loops at high relative pressure. It is proved that adsorbents have the properties of mesoporous material. The pore size distribution calculated by the BJH method is shown in Fig. 6b. Compared with MgAl-LDH, γ-AlO(OH) and γ-AlO(OH)/MgAl-LDH have wider pore size distribution curves. As shown in Table 1, BET analysis results of MgAl-LDH, γ-AlO(OH), and γ-AlO(OH)/MgAl-LDH were 14.1 m^2^ g^−1^, 95.9 m^2^ g^−1^, and 34.1 m^2^ g^−1^, respectively. The results demonstrated that the excellent adsorption performance of γ-AlO(OH)/MgAl-LDH does not depend on the large specific surface area. The optical photographs of the samples before and after adsorbing for MO are shown in Fig. 6c (before centrifugation). According to the optical photographs before adsorption, the original volume of the sample was γ-AlO(OH)/MgAl-LDH < γ-AlO(OH) < MgAl-LDH. However, after the adsorption of MO (1000 mg L^−1^), it was obvious that the volume of powders has changed. The volume expansion rate of the samples was as follows: γ-AlO(OH)/MgAl-LDH > γ-AlO(OH) > MgAl-LDH. Therefore, it could be inferred that the volume expansion rate of the adsorbent has a great influence on the adsorption performance of MO.

**Fig. 7:**
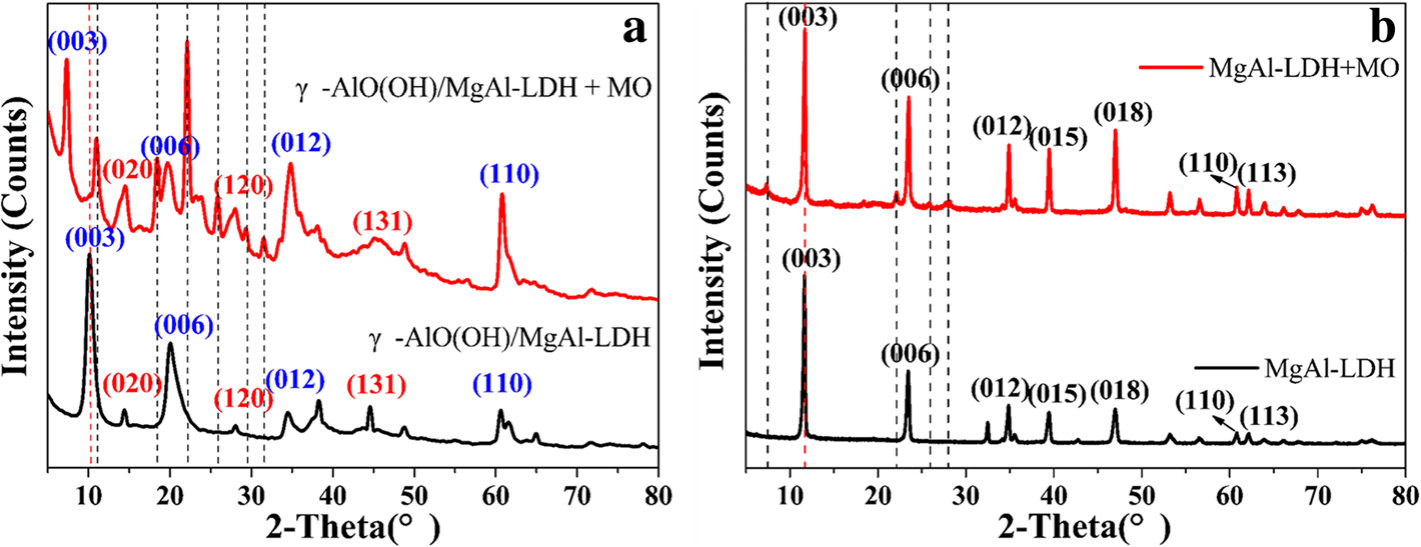
The XRD patterns of γ-AlO(OH)/MgAl-LDH (**a**) and MgAl-LDH (**b**) before and after adsorbed MO, respectively

**Scheme 1:**
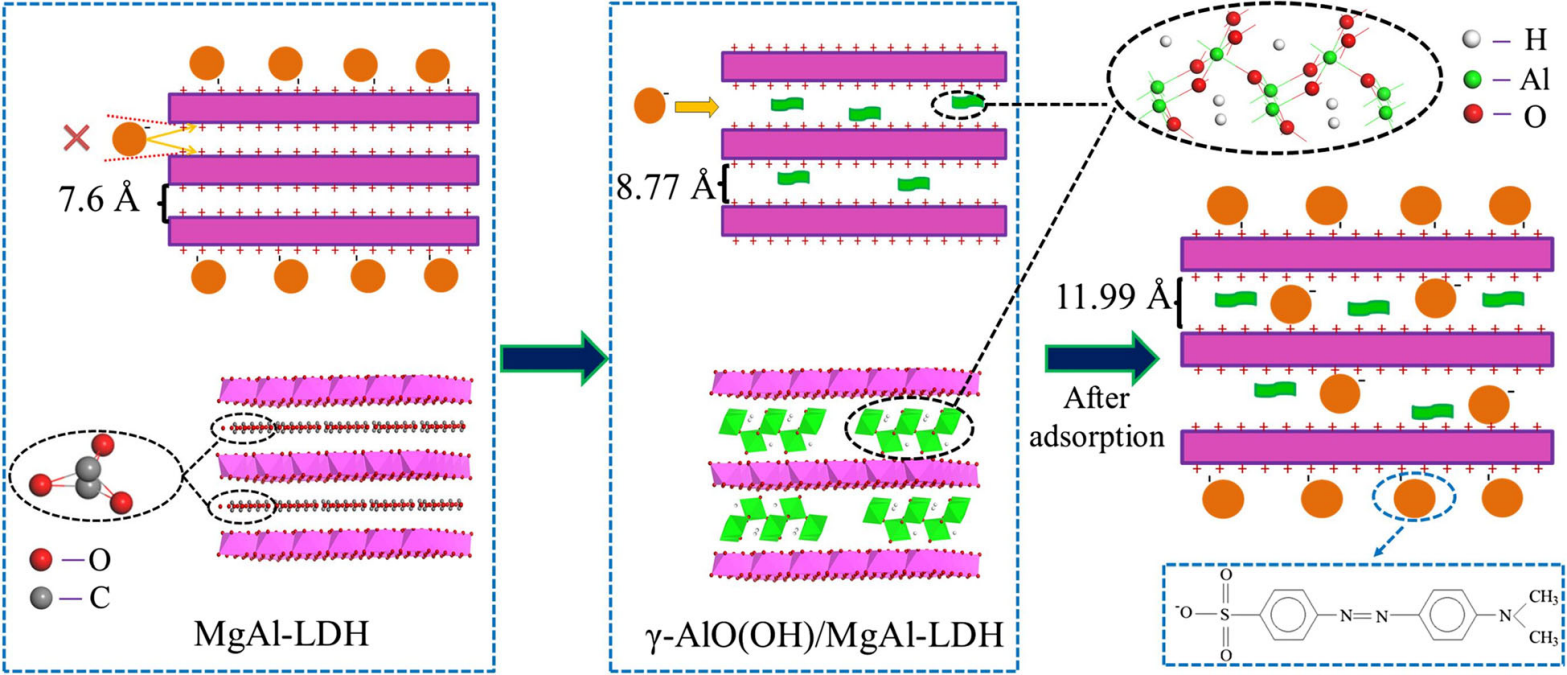
The mechanism of γ-AlO(OH)/MgAl-LDH for enhancing adsorption of MO

Figure 7a shows the XRD patterns of γ-AlO(OH)/MgAl-LDH before and after MO adsorption. Compared with γ-AlO(OH)/MgAl-LDH, many new peaks appeared in the spectra of γ-AlO(OH)/MgAl-LDH after MO adsorption. In addition, except for the (003) and (006) planes of MgAl-LDH, the other planes were not shifted to a low angle. The new peaks indicate that the MO anions enter the interlayer of γ-AlO(OH)/MgAl-LDH via anion exchange and lead to a shift of the (003) plane to the minor angle plane [8]. More importantly, the (003) plane of γ-AlO(OH)/MgAl-LDH increased by 3.22 Å from 8.77 to 11.99 Å after MO adsorption. Interestingly, as shown in Fig. 7b, the degree corresponding to the (003) plane of MgAl-LDH did not change after adsorbing MO, indicating that MO cannot be adsorbed in MgAl-LDH layers. It is observed that γ-AlO(OH) plays an important role in the LDH layer, and the adsorption effect on MO is shown in Scheme 1. On the one hand, by the “space confined” effect, nanoneedle γ-AlO(OH) can be grown between the MgAl-LDH layers to expand the (003) plane spacing, which helps MO to enter the MgAl-LDH intermediate layer by electrostatic attraction. On the other hand, MgAl-LDH has more space for storing MO, due to the expansion between the LDH layers.

**Fig. 8:**
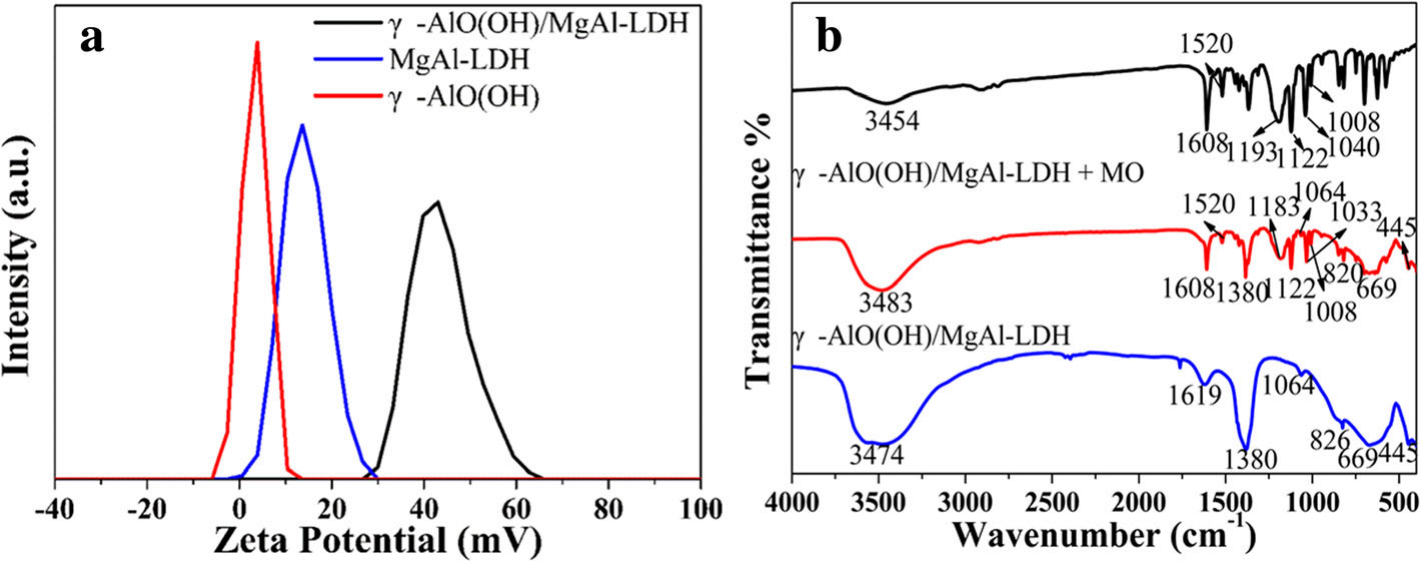
ζ-potentials of samples (0.2 mg mL^−1^) in aqueous solution at pH = 3 (**a**). FTIR spectra of MO, γ-AlO(OH)/MgAl-LDH before and after adsorbed MO (**b**)

From the above discussion, it is known that γ-AlO(OH)/MgAl-LDH has a higher adsorption kinetics and adsorption performance than γ-AlO(OH) and MgAl-LDH. To better study the mechanism of performance improvement, the samples were subjected to a zeta potential test. As shown in Fig. 8a, the suspension of the samples at pH = 3 showed a positively charged surface and the ζ-potential value of γ-AlO(OH)/MgAl-LDH (43.03 mV) was much greater than that of MgAl-LDH (13.88 mV) and γ-AlO(OH) (4.32 mV). This result indicates that the synergistic effect can be produced due to γ-AlO(OH) entering the MgAl-LDH layer, which improves the zeta potential of the γ-AlO(OH)/MgAl-LDH sample. As shown in Scheme 1, the MO molecule can be formed to C_14_H_14_N_3_SO_3_^−^ and Na^+^ in water. Because C_14_H_14_N_3_SO_3_^−^ anion is negatively charged, it is easily to be adsorbed by γ-AlO(OH)/MgAl-LDH. It could be inferred that LDH presents a good adsorption capacity for anionic dyes.

The FTIR spectra of γ-AlO(OH)/MgAl-LDH after adsorbing MO is shown in Fig. 8b. Compared with the original γ-AlO(OH)/MgAl-LDH, the FTIR spectra of γ-AlO(OH)/MgAl-LDH showed obvious changes after absorbing MO. The bands at 1608 and 1520 cm^−1^ were due to the N=N stretching vibration and the N–H bending vibration. In addition, the bands at 1183 and 1033 cm^−1^ were due to the asymmetric and symmetric stretching vibrations of the sulfonic acid group (–SO_3_^−^), respectively. The band at 1122 cm^−1^ was due to the symmetrical stretching vibration of O=S=O [36]. The band at 1008 cm^−1^ was related to the C–H aromatic in-plane bending vibration [1]. Obviously, the O–H peak of γ-AlO(OH)/MgAl-LDH was shifted from 3474 to 3843 cm^−1^ when MO was absorbed, indicating that hydrogen bonding participates in the adsorption process.

**Fig. 9:**
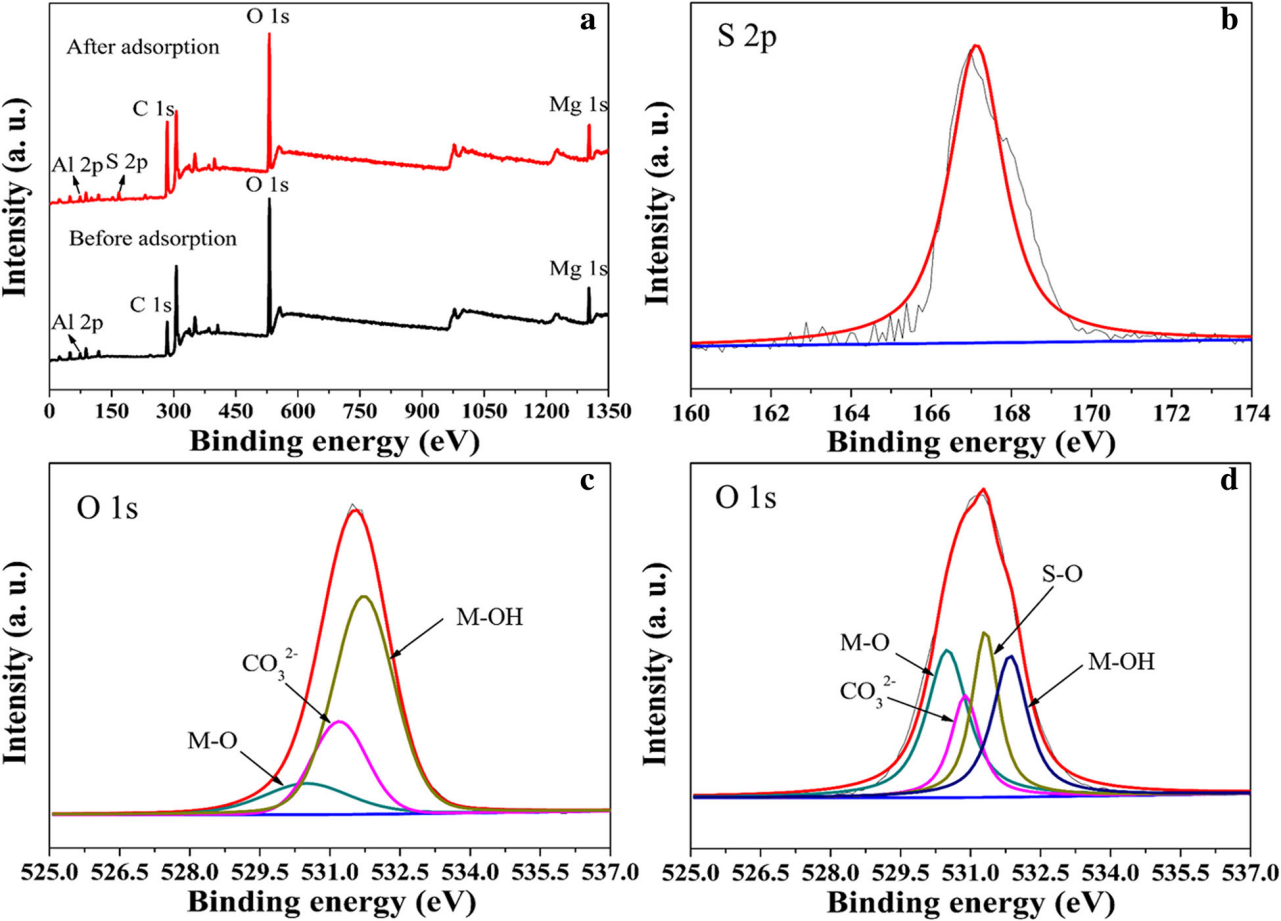
Wide XPS spectra of γ-AlO(OH)/MgAl-LDH before and after MO adsorption (**a**), S 2p narrow XPS of γ-AlO(OH)/MgAl-LDH after adsorbed MO (**b**), O 1s spectrum of γ-AlO(OH)/MgAl-LDH (**c**), O 1s spectrum of γ-AlO(OH)/MgAl-LDH after adsorbed MO (**d**)

In addition, XPS was used to characterize the adsorbent before and after adsorbing MO. As shown in Fig. 9, the S element appeared in the spectrum of γ-AlO(OH)/MgAl-LDH after adsorbing MO. The high-resolution spectrum of S 2p located at 167 eV, shown in Fig. 9b, indicates the presence of MO in the adsorbent. Figure 9c, d shows the O 1s spectrum of γ-AlO(OH)/MgAl-LDH before and after MO adsorption, respectively. Three peaks located at 530.5, 531, and 531.8 eV are shown in Fig. 9c, d, and can be assigned to the O in the forms of metal oxide (M–O), the carbonate (CO_3_^2−^), and metal-hydroxyl (M–OH) of the MgAl-LDH interlayer [1]. Notably, there are significant changes in the intensity and composition of O 1s of the adsorbent after MO adsorption. The newly appearing peak located at 531.6 eV in Fig. 9d could be assigned to the O in the sulfate group (S–O). In addition, as shown in Table 5, the CO_3_^2−^ decreased from 27.2 to 18.1%, due to ion exchange. The relative proportion of M–O after adsorbing MO is increased from 9 to 26.4%, and the relative proportion of M–OH decreased from 63.8 to 25.7%. The results show that the hydroxyl active site of γ-AlO(OH)/MgAl-LDH plays an important role in the adsorption of methyl orange, indicating that adsorption of MO is controlled by chemical interactions; this is consistent with the pseudo-second-order kinetic model.

**Table 5:** Distribution of components from XPS spectra of O 1s peak of adsorbent before and after MO adsorption

Samples	Peak	Binding energy (eV)	Percent (%)
γ-AlO(OH)/MgAl-LDH	CO_3_^2−^	531	27.2
	M–O	530.5	9
	M–OH	531.8	63.8
γ-AlO(OH)/MgAl-LDH after MO adsorption	CO_3_^2−^	531	18.1
	M–O	530.5	26.4
	M–OH	531.8	25.7
	S–O	531.6	29.8

### Adsorbent Recycling

**Fig. 10:**
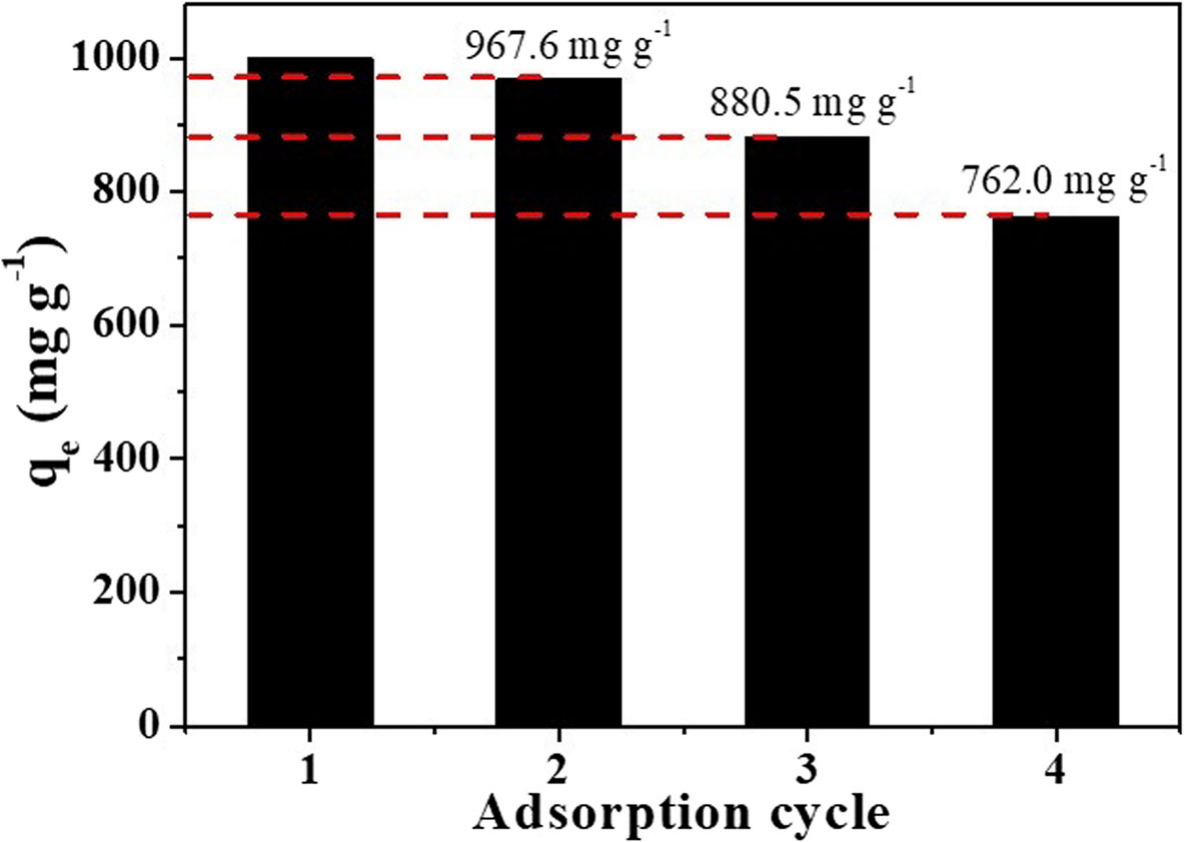
Adsorption cycle performance of γ-AlO(OH)/MgAl-LDH. Initial MO concentration: 1000 mg L^−1^

The regeneration performance of the γ-AlO(OH)/MgAl-LDH adsorbent was studied via adsorption-desorption cycles. As shown in Fig. 10, the adsorption capacity of γ-AlO(OH)/MgAl-LDH remained at 762 mg g^−1^ after 4 cycles, and the removal efficiency remained above 76%. The decrease in capacity is due to the incomplete desorption of MO and the loss of the adsorbent during the adsorption and washing of the dye molecules. These results indicate that γ-AlO(OH)/MgAl-LDH can be considered an efficient and recyclable adsorbent for the removal of MO from water.

## Conclusion

The composite of γ-AlO(OH)/MgAl-LDH was synthesized by one-pot method. As an adsorbent, the composite exhibits an excellent adsorption performance for MO. On the one hand, based on the “space-confined” effect, γ-AlO(OH) nanoneedle was prepared between the MgAl-LDH layers. The expansion between the LDH layers leads to more space for storing MO. On the other hand, the hydroxyl active site results in a chemical interaction between γ-AlO(OH)/MgAl-LDH and MO, which promotes the adsorption kinetics. Therefore, the γ-AlO(OH)/MgAl-LDH exhibits an excellent adsorption performance for MO, which can be completely adsorbed in 210 min at the initial concentration of 1000 mg L^−1^. After 4 cycles, the regenerated adsorbent can maintain an initial adsorption capacity of more than 76%. In addition, the maximum adsorption capacity of γ-AlO(OH)/MgAl-LDH reaches 4681.40 mg g^−1^ according to the Langmuir adsorption model. Based on the positive zeta potential of γ-AlO(OH)/MgAl-LDH, the composite has stronger adsorption kinetics and adsorption properties for anionic dyes such as MO, Congo Red, and Acid Orange 7. These adsorbed dyes can be desorbed and reutilization, or incinerated directly. In addition, the composite is also a potential photocatalyst carrier. When the photocatalyst is loaded on γ-AlO(OH)/MgAl-LDH, the dyes will be rapidly absorbed around the catalyst, which improves the photocatalytic reaction kinetics. Therefore, γ-AlO(OH)/MgAl-LDH has great potential in water pollution treatment.

## Data Availability

The datasets generated during and/or analyzed during the current study are available from the corresponding author on reasonable request.

## Abbreviations

DDS: Organic anion dodecylsulfate
DI: Deionized
FESEM: Field-emission scanning electron microscopy
FTIR: Fourier-transform infrared spectroscopy
HRTEM: High-resolution transmission electron microscopy
LDHs: Layered double hydroxides
MO: Methyl orange
TEM: Transmission electron microscopy
XPS: X-ray photoelectron spectroscopy
XRD: X-ray powder diffraction
γ-AlO(OH): Aluminum oxide hydroxide
